# Effect of Pressure and Temperature on Densification in Electric Field-Assisted Sintering of Inconel 718 Superalloy

**DOI:** 10.3390/ma14102546

**Published:** 2021-05-13

**Authors:** Liyong Ma, Ziyong Zhang, Bao Meng, Min Wan

**Affiliations:** 1 School of Mechanical Engineering and Automation, Beihang University, Beijing 100091, China; maliyong@buaa.edu.cn (L.M.); ZY1907321@buaa.edu.cn (Z.Z.); mwan@buaa.edu.cn (M.W.); 2 School of Mechanical Engineering, Hebei University of Architecture, Zhangjiakou 075000, China

**Keywords:** electric field-assisted sintering, Inconel 718 superalloy, FKM-GTN model, pressure, temperature, densification

## Abstract

Electric field-assisted sintering has ubiquitous merits over conventional sintering technology for the fabrication of difficult-to-deform materials. To investigate the effect of sintering pressure and temperature on the densification of Inconel 718 superalloy, a numerical simulation model was established based on the Fleck-Kuhn-McMeeking (FKM) and Gurson-Tvergaard-Needleman (GTN) models, which covers a wide range of porosity. At a sintering pressure below 50 MPa or a sintering temperature below 950 °C, the average porosity of the sintered superalloy is over 0.17 with low densification. Under a pressure above 110 MPa and a temperature above 1250 °C, the sintered superalloy quickly completes densification and enters the plastic yield stage, making it difficult to control the sintering process. When the pressure is above 70 MPa while the temperature exceeds 1150 °C, the average porosity is 0.11, with little fall when the pressure or temperature rises. The experimental results indicated that the relative density of the sintered superalloy under 70 MPa and 1150 °C is 94.46%, and the proportion of the grain size below 10 μm is 73%. In addition, the yield strength of the sintered sample is 512 MPa, the compressive strength comes to 1260 MPa when the strain is over 0.8, and the microhardness is 395 Hv, demonstrating a better mechanical property than the conventional superalloy.

## 1. Introduction

Inconel 718 superalloy is a nickel-chromium-iron-based superalloy, which is widely used in aviation engines, nuclear power and other precision equipment and instruments due to its good fatigue, creep properties and corrosion resistance under extreme temperatures and pressures [1,2]. Forging, casting [3] and sintering [4] are the main forming methods for Inconel 718, among which, the sintering technology can effectively save energy and raw materials. Moreover, the sintered parts have good mechanical properties and precision [5].

In recent years, electric field-assisted sintering technology has been widely applicated in the field of powder metallurgy, effectively accelerating the densification process, shortening the sintering time, reducing the sintering temperature and improving production efficiency. In addition, the electric field-assisted sintering can also suppress the growth of crystal grains and contribute significantly to the integrated control of workpiece shape and performance [6]. Elder Soares et al. [7] employed spark plasma sintering (SPS) to prepare dense AA7075 high-strength aluminum alloy in a one-step process under the sintering temperature of 550 °C, and the uniaxial pressure of 100 MPa, and the heat preservation time was 15 min. Due to the low sintering temperature and short sintering time, the satisfying grain size and morphology in the sintered sample were retained. Yu. NP et al. [8] obtained AlMgB14-based materials through SPS. The density of the sample sintered at the temperature of 1400 °C was 2.621 g/cm^3^ (the theoretical density of pure AlMgB14 is 2.59 g/cm^3^), and the mechanism of the decomposition of AlMgB14 was proposed when the (Al-Mg)-B powder mixture was locally overheated in the SPS process. Mahundla M R et al. [9] sintered Ti, Ti-6Al-4V (Ti-64) and Ti-34Nb-25Zr (TNZ) via SPS, with the sintering temperature of 1200 °C, the sintering pressure of 50 MPa, the heating rate of 100 °C/min and the heat preservation time of 10 min. The densification degrees of the three alloys were between 97% and 100%, and the Vickers microhardness values of the samples were 371.41, 385.48 and 590.22 HV, respectively. In terms of electric field-assisted sintering of superalloys, Cartón-Cordero M et al. [10] sintered cobalt-based superalloy Co-9Al-9W by electric field-assisted hot pressing. After sintering, the cobalt-based superalloy obtained a fine grain structure with a *γ*/*γ*’ microstructure, and the compressive strength was increased by 45%. Yan S J et al. [11] prepared Inconel 718 superalloy through SPS and plasma rotating electrode process, followed by solution aging treatment. The densification rate of the Inconel 718 superalloy after SPS was 97%, and the yield strength was 419 MPa. In addition, the Granger and Guide models were employed to reveal the densification kinetics related to the deformation rate of the powder particles in the SPS process.

The densification response in the electric field-assisted sintering is closely related to the coupling effect of the multi-energy field parameters and the three-dimensional morphology (such as grain and pore distribution, etc.) of the sintered sample. Many macro and micro mechanisms have been proposed to explain the densification process of the powder under the influence of electric current and pressure, but the densification state of the sintered sample cannot be timely observed during the sintering process. In addition, the internal temperature can only be measured locally on the outer wall of the mold with a pyrometer whose thermocouple has an upper detecting limit that stints the measurement of high temperature, making it difficult to predict the final microstructure and its relationship with the sintering parameters. Therefore, an effective numerical simulation for electric field-assisted sintering of superalloys is necessary to capture the process parameters of multi-physics, and to solve the problem of the coupling effect of each parameter in the nonlinear complex multi-physics on the densification of superalloy, which is of great significance to the coordinated control of the shape and the properties during the sintering process. At present, the numerical simulation of multi-energy field coupling sintering mainly uses molecular dynamics, discrete elements, Monte Carlo dynamics, etc. Molecular dynamics models [12,13] can calculate the atomic-level mass transmission during the sintering process, however, the simulating sintering time is short (usually nanoseconds), and the simulated size is limited to nanometers. The discrete element method [14,15] regards the sintered particles as independent ones, and analyses the evolution process of mechanical and microstructure properties by calculating their interaction during the particle aggregation process. However, due to the simplified size of the sintering neck, the discrete element method is not suitable for relatively dense sintering systems. Monte Carlo dynamics model [16,17,18] is based on the discrete element method, through the layered study of the sintered sample, captures the microstructure evolution from the initial stage to the final stage of sintering, and can observe the grain size and shape. Monte Carlo dynamics model regards pore migration, radiation effect, relative density, grain boundary energy, curvature and surface energy as core sintering parameters. This method can process hundreds of non-spherical particles. Combined with the use of X-ray diffraction (XRD) technology to quantitatively test the microstructure, it can reproduce the sintering track of the powders. Even though the Monte Carlo kinetic model can explain the densification process of each sintered area layer by layer, the number of layers is limited and the layers are discrete. As a result, the effect of the temperature gradient and pressure change on the continuous process of powder densification cannot be fully revealed.

In 1977, Gurson [19] established a constitutive equation for porous plastic materials, which took the porosity as an internal parameter, was applied to porous plastic materials with low porosity. When it came to the yield condition and damage evolution of the Gurson model, Tvergaard and Needleman [20,21,22] added the empty core law for further modification. Therefore, this equation is called the Gurson-Tvergaard-Needleman (GTN) model. In 1992, Fleck, Kuhn and McMeeking [23] applicated the modified GTN model in the plastic yield process of powder sintering and established the Fleck-Kuhn-McMeeking (FKM) constitutive model, which believed that the powder particles were joined together by contact, and then the contact interface and the nearby materials were plastically deformed to produce the corresponding deformation. Therefore, the sintered powders can be regarded as an isotropic and homogeneous porous continuum in a macroscopic view. Meanwhile, the macroscopic effects of deflection strain and volume change are also considered. When the heterogeneous powder material is assumed to be macroscopically uniform, the continuous field theory can be employed [24]. In the continuous field theory, the porosity has a linear relationship with the relative density, which determines the degree of densification of the sintering process. In addition, heat capacity, thermal expansion, electric current and thermal conductivity vary with the fluctuation of temperature, relative density and porosity. Therefore, the porosity is the key parameter for the electro-thermal-force multi-field coupling model.

In this study, the FKM model was combined with the porous plastic GTN model to establish a numerical simulation model for characterizing the electric field-assisted sintering process of Inconel 718 superalloy covering a larger range of porosity, which was used to analyze the densification progress of the metal powder sintering process and the geometric nonlinearity caused by large displacement. Through the numerical simulation, we mainly focused on the effects of sintering pressure and temperature on the densification of Inconel 718 alloy. In addition, the validity of the established model and numerical simulation was verified through electric field-assisted sintering experiments.

## 2. Theoretical Basis of Electric Field-Assisted Sintering

### 2.1. FKM-GTN Model

When the superalloy powder is in the process of sintering, the relationship between porosity and relative density can be expressed as:

(1) ϕ=1−D

where *ϕ* is the porosity and *D* is the relative density. When the porosity *ϕ* is higher than 0.25, the sintered sample lies in the early stage of sintering and obeys the FKM yield model [24]:

(2) $$\Phi_{F}=\frac{2(1-\phi )}{\phi^{2}}\cosh\left(\frac{2\Sigma_{m}}{\sigma_{S}}\right)+{\left(\frac{\Sigma }{\phi \sigma_{S}}\right)}^{2}-\frac{2(1-\phi )}{\phi^{2}}-1=0$$

where *Φ_F_* is the plastic potential of the FKM model, *Σ* is the Mises stress, *Σ_m_* is the hydrostatic stress on the mesoscopic scale, and *σ_s_* is the yield stress of the sintered sample.

In the early stage of sintering, the growth of nucleus and shear significantly affect the change of porosity Δ*ϕ*:

(3) $$\Delta \phi =\Delta \phi_{C}+\Delta \phi_{S}$$

where $\Delta \phi_{C}=\frac{D}{S_{N}\sqrt{2\pi }}\varepsilon e^{\frac{1}{2}(\frac{\varepsilon -\varepsilon_{N}}{s_{N}})}$ is the increase of porosity when crystal nuclei grow, Δ*ϕ_s_* = *k_w_ϕn^D^ε* is the increase of porosity accompanying the shear growth, *ε_N_* is the nucleus strain, *S_N_* is the standard deviation, *k_w_* is the material shear parameter, *n^D^* is the deflection tensor coaxial with the stress tensor, *w* is the stress tensor and *ε* is the plastic strain rate that depends on the porous plastic model. The relationship between *ε* and *Σ* is

(4) Σ=E(ε−λg)

where $g=\frac{\partial \Phi }{\partial \Sigma }$, *E* is the modulus of elasticity, and *λ* is a non-negative multiplier, satisfying the Kuhn-Tuker condition [25]:

(5) λΦ=0,λ≥0

The relative density *D* and the plastic strain rate *ε* satisfy the following differential equation:

(6) $$\dot{D}=-D\cdot \frac{\partial \varepsilon }{\partial V}$$

where *V* is the volume of the pre-sintered sample. Therefore, according to Equations (1) and (6), the reducing rate of porosity is controlled by plastic strain, as shown in Equation (7):

(7) $$\dot{\phi }=(1-\phi )\cdot \frac{\partial \varepsilon }{\partial V}$$

When the porosity is below 10%, the plastic deformation in the sintered sample obeys the GTN model [25]. The yield equation and plastic potential of the GTN model can be expressed as:

(8) $$\Phi_{G}=2q_{1}\phi \cdot \cosh\left(\frac{3q_{2}\Sigma_{m}}{2\sigma_{s}}\right)+{\left(\frac{\Sigma }{\sigma_{s}}\right)}^{2}-1-q_{3}\phi^{2}=0$$

where *Φ*_G_ is the plastic potential of the GTN model, and *q*_1_, *q*_2_, *q*_3_ are the Tvergaard correction coefficients employed by Tvergaard [20] on the basis of the Gurson damage model, which are used to characterize the interaction between adjacent holes during material deformation. According to Tvergaard’s suggestion [20,21], the value of *q*_1_, *q*_2_ and *q*_3_ should be 1.5, 1 and 2.25 for the metallic material, respectively. When the porosity is between 10% and 25%, which is a transitional state, a linear combination of both models is usually expressed as Equation (9):

(9) $$\Phi =\mu_{G}\Phi_{G}+\mu_{F}\Phi_{F}=0$$

where *μ*_G_ and *μ*_F_ are the scale coefficients in the GTN and the FKM models in the linear combination, respectively, and *μ*_G_ + *μ*_F_ = 1.

### 2.2. Electric Field Model

The graphite die and the superalloy powder are connected in series in the electric field system, and the electric current density *J* is determined by Maxwell’s equation:

(10) $$J=\lambda \left(D,T\right)\cdot E$$

where ***E*** is the electric field intensity vector, ***E* =** −∇*U*, *U* is the electric potential, *λ* is the conductivity and *T* is the sintering temperature, and [*λ*(*ϕ*,*T*)·***E***] = 0.

According to Joule’s law, when the electric current passes through the graphite mold and superalloy powder, the generated Joule heat *Q* is expressed as:

(11) $$Q=\int_{V}J\cdot EdV=\int_{V}\frac{J^{2}}{\lambda (D,T)}dV$$

where *V* is the volume of the conductor. It is seen from Equations (10) and (11) that the electric and the thermal field can be coupled through the conductivity *λ*, which determines the relative density and the sintering temperature.

### 2.3. Heat Transfer and Thermal Expansion Model

According to the heat transfer theory, the temperature distribution of Joule heat in the sintered sample obeys the Fourier heat balance equation:

(12) $$DC\frac{\partial T}{\partial t}=\frac{1}{r}\left[\frac{\partial }{\partial z}\left(kr\frac{\partial T}{\partial z}\right)\right]+\frac{\partial }{\partial r}\left(kr\frac{\partial T}{\partial r}\right)+DL\left(\frac{\partial f_{S}}{\partial T}\cdot \frac{\partial T}{\partial t}\right)+\frac{J^{2}}{\lambda (D,T)}$$

where *C* is the specific heat capacity, *z* is the longitudinal coordinate of the polar coordinate system, *r* is the radial coordinate of the polar coordinate system, *k* is the thermal conductivity, *f_S_* is the mass fraction of the solid phase and *L* denotes the latent heat of phase change.

(13) $$L=\left(C-C_{e}\right)/\frac{\partial f_{S}}{\partial t}$$

where *C_e_* is the effective specific heat capacity.

During the electric field-assisted sintering process, the porous domain develops from a loosely packed state (*ϕ* = 0.4) to an almost fully dense state. The instantaneous relative density and axial compression of the sintered sample must satisfy the conservation of volume:

(14) $$\frac{D}{D_{0}}=\frac{V_{0}}{V}=\frac{\delta_{0}}{\delta }$$

where *δ* is the axial compression and the subscript “0” represents the initial state. Correction is conducted in the displacement *s* of the punch according to the thermal expansion effect, then the compression *δ* of the sintered sample can be measured by the displacement *s* of the punch:

(15) $$\delta =s-\delta_{T}$$

where *δ_T_* is the axial thermal expansion of the sintered sample, *δ_T_* = *α*(*T* – *T*_0_), and *α* is the thermal expansion coefficient, which can be calculated in Ref. [26].

## 3. Numerical Simulation

### 3.1. Material and Process

**Figure 1:**
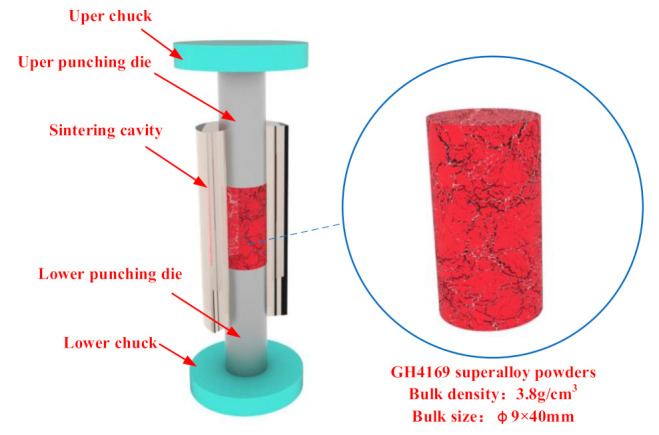
Die structure and the Inconel 718 power.

Inconel 718 nickel-based superalloy was adopted as the powder material, the punching die material was graphite and the sintering cavity was a quartz tube. The die structure is shown in Figure 1. The sintering cavity contained loosely packed Inconel 718 nickel-based superalloy powder. The upper and lower cylindrical dies were inserted into the upper and lower ends of the sintering cavity, respectively, and the Inconel 718 superalloy was pre-pressed, the axis of which coincided with that of the sintering cavity. The outer ends of the upper and lower punches were inserted into the center hole of the conductive chuck to transmit the applied load and electric current. According to the FKM-GTN theoretical model, the properties of superalloy powder and the die material, the numerical simulation parameters are set as shown in Table 1.

### 3.2. Numerical Simulation Process

The multi-physics finite element analysis software COMSOL Multiphysics(Version 5.4, COMSOL Co., Ltd., Stockholm, Sweden) was used to carry out the multi-physics coupling numerical simulation on electric field-assisted sintering. Since the sintering cavity, die and powder were all cylindrical axisymmetric bodies, the numerical model was simplified to a two-dimensional axisymmetric model. According to the FKM-GTN model, the powders were regarded as a porous plastic continuum, and the upper and lower end faces of the pre-pressed sample were in contact with the inner end faces of the upper and lower punching dies. The sample was set to conduct electricity, heat and solid mechanics. As a thermally and electrically insulated contact surface, the side face contacted the inner surface of the sintering cavity, and the sintering pressure was applied to the outer end faces of the upper and lower dies. The electric potential at the bottom end of the lower die was 0, meanwhile, the electric potential of the top end of the upper die was the highest, and electric current could pass through the punch and the powder. The two-way compression method was adopted, the applied sintering pressure range was 50~110 MPa, the temperature was 950~1250 °C and the heating rate was 300 °C/min. The simulation scheme is shown in Table 2.

**Figure 2:**
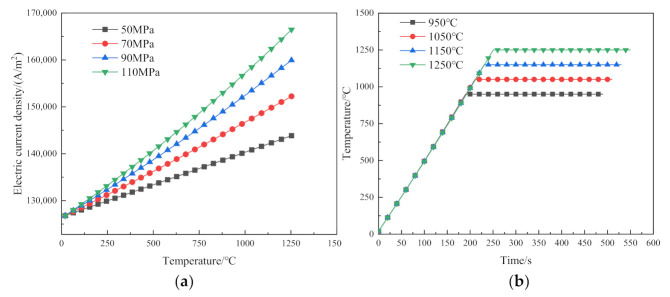
The relationship between electric current density and sintering temperature: (**a**) Setting trajectory of electric current density at each target sintering temperature; (**b**) variation of sintering temperature with time.

To ensure that the given voltage and the temperature rise rate in each sintering experiment are the same, when the sintering pressure changes, according to Equation (10), the applied electric current density should be accordingly changed. Due to the small sintering stroke per unit time, the resistance change of the porous plastic continuum model of the metal powders is slow when the sintering pressure is low. Therefore, the rate of change of current density with temperature is small, and vice versa. The setting trajectory of the electric current density at each target sintering temperature is shown in Figure 2a. Under the influence of the electric current, the heating rate of the sintering temperature is the same, and its variation with time is shown in Figure 2b.

## 4. Results and Discussion

### 4.1. Effect of Sintering Pressure on the Densification of Inconel 718 Superalloy

**Figure 3:**
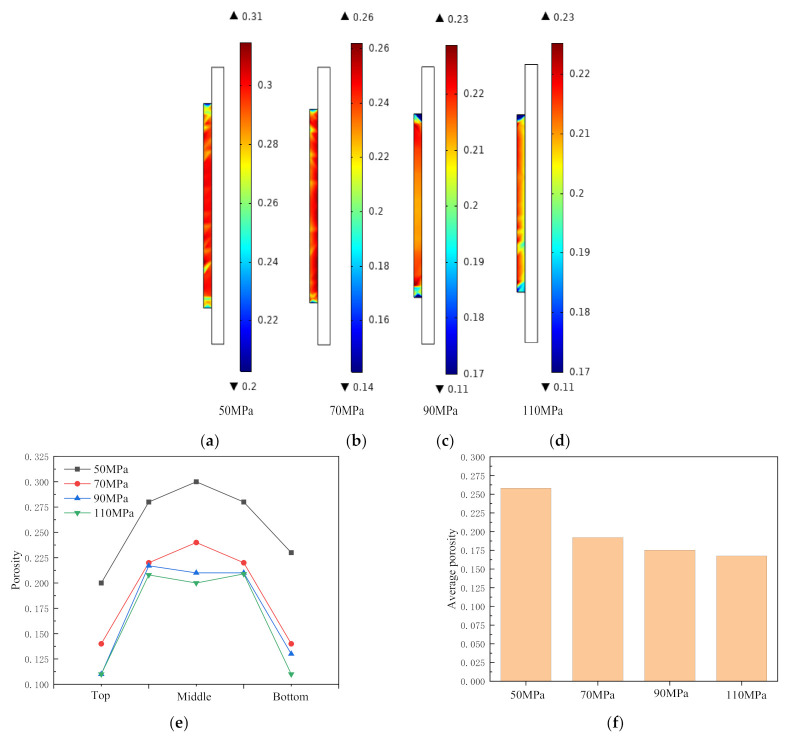
Influence of sintering pressure on porosity of sintered sample at 950 °C: (**a**) 50 MPa; (**b**) 70 MPa; (**c**) 90 MPa; (**d**) 110 MPa; (**e**) porosity distribution under different pressures at 950 °C; (**f**) average porosity under different pressures at 950 °C.

The porosity distribution of the sintered samples with the pressures of 50 MPa, 70 MPa, 90 MPa and 110 MPa at 950 °C is shown in Figure 3. Comparing Figure 3a–d, the overall porosity presents a lower top and bottom part, and a higher middle part distribution trend. It can be seen from Figure 3a,f that the average porosity under 50 MPa is the largest, and the maximum porosity is 0.3 in the middle, which is only 0.1 lower than the initial porosity. According to Figure 3e, the whole sintered sample completely presents a “Λ”-shaped porosity distribution with low middle and high sides. The porosity at this time is greater than the critical porosity for plastic flow [27],

(16) $$\phi_{\lim}=\exp(-3\sigma /2\sigma_{s})$$

where *ϕ*_lim_ is the critical porosity, *σ* is the stress and *σ_s_* is the yield stress.

According to Equation (16), the densification process of the sintered sample at this stage is mainly plastic flow. After the porosity drops to a certain value, it remains unchanged. According to Figure 3b–d, the average porosity under the three sintering pressures of 70 MPa, 90 MPa and 110 MPa is close. The porosity of the middle part of the sample after sintering under the condition of 70 MPa begins to decrease, and the porosity of the middle part under the conditions of 90 MPa and 110 MPa is decreased. As shown in Figure 3e, under the three sintering pressures, the porosity of the sintered sample from both ends to the middle presents an “M”-shaped distribution, which indicated that the plastic flow in the middle area of the sintered sample is close to saturation, but the powders at the top and end move to the inside, demonstrating an uncompleted large-strain plastic flow with large pores kept in the sintered sample. When the sintering pressure is increased to 110 MPa, the average porosity of the sample after sintering is the smallest, and the minimum porosity is 0.08, which is located on a small area on the top of the sample. The porosity of other areas tends to be uniform, around 0.2, failing to meet the densification requirements. According to Figure 3f, the average porosity under each sintering pressure is higher than 0.17, indicating a relatively low degree of densification.

**Figure 4:**
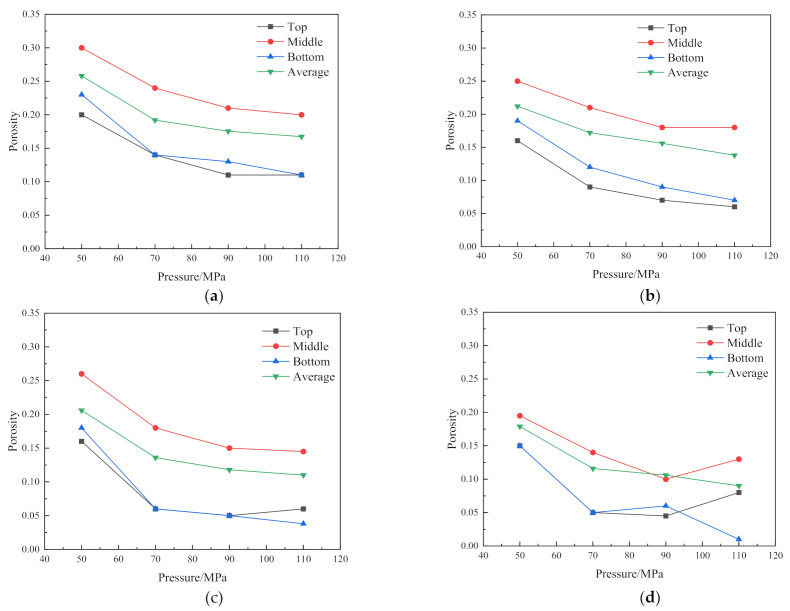
Effect of sintering pressure on porosity under various sintering temperatures: (**a**) 950 °C; (**b**) 1050 °C; (**c**) 1150 °C; (**d**) 1250 °C.

To study the effect of sintering pressure on the densification of the sintered sample, sintering pressures of 50 MPa, 70 MPa, 90 MPa and 110 MPa are applied to observe the trend of changing in the top, middle and bottom, respectively, and average porosity of the sintered samples. Figure 4a–d are the porosity curves of the sintered sample under the sintering pressures of 50 MPa, 70 MPa, 90 MPa and 110 MPa and the temperature of 950 °C, 1050 °C, 1150 °C and 1250 °C, respectively.

Figure 4a shows the variation trend of the porosity distribution of the sample with the sintering pressure under the condition of 950 °C. With the increase of the sintering pressure, the porosity e decreases gradually. The porosity of the two ends of the sintered sample is lower than that of the middle part, and the minimum average porosity is 0.167. It shows that the large strain plastic flow from the two ends to the middle has not been completed, and the large pores still exist.

Figure 4b depicts the changing trend of porosity of the sintered samples with the variation of pressures at 1050 °C. The porosity distribution under 50 MPa is extremely uneven, the average porosity is about 0.21 and the degree of densification is low. The porosity of the sintered sample under the condition of 70 MPa tends to be uniform. It can be seen from Figure 4b that the porosity of 90 MPa and 110 MPa has reached below 0.16, the difference between the average porosity and the porosity in the middle of the sintered sample reduces, indicating that the densification reaches a higher level. These phenomena all indicate that after the large strain plastic flow in the central region approaches saturation, the plastic flow from the top to the bottom is almost completed, and the large pores inside the sintered sample are eliminated and begin to enter a steady state. The densification is mainly caused by the co-domination of dislocation creep and diffusion creep [25]. 

Figure 4c gives the porosity distribution of the sintered samples under the sintering pressures of 50 MPa, 70 MPa, 90 MPa and 110 MPa at 1150 °C, respectively. The distribution of porosity under the pressure of 50 MPa is similar to Figure 4a, indicating that the densification under this condition is still dominated by the plastic flow. In addition, the average porosity is 0.2, showing the degree of the densification is low. When the sintering pressure is 70 MPa, 90 MPa and 110 MPa, the average pores of the sintered samples are all reduced to below 0.15, indicating that the secondary densification dominated by dislocation creep and diffusion creep has completely developed from the middle to the ends after the large pores inside the sintered sample are eliminated.

It is seen from Figure 4d that when the sintering pressure is 50 MPa, the degree of densification of the sintered sample is much higher than that under 1150 °C and 50 MPa, and the difference in porosity among each part is small. When the sintering pressure is low, increasing the sintering temperature can also achieve relatively low porosity, but the degree of densification is not high. When the sintering pressure is 70 MPa and 90 MPa, the porosity decreases with the increase of the pressure, and the average porosity reaches below 0.13. Especially at 90 MPa, the average porosity of the sintered sample is the same as the middle porosity, reaching about 0.1, indicating the sintered sample reaches a higher degree of densification. It is worth noting that the porosity of the top and middle of the sintered sample undergoes abrupt changes when the pressure is increased to 110 MPa. Compared with 90 MPa, the degree of densification of the sintered sample under the condition of 110 MPa is worse. The main reason for these phenomena is that under high temperature and high pressure, the densification is completed quickly and the completely dense sintered body continues to bear greater pressure, and yield deformation begins to appear inside. Therefore, the sintering pressure above the temperature of 1250 °C and above 110 MPa makes the sintered body rapidly complete densification and enter a state of plastic deformation, which makes it very difficult to control the sintering process under this condition. In addition, it can be seen from the Figure 4d that when the sintering pressure reaches 70 MPa, although the average porosity of the sintered sample increases with the increase of pressure, the change is not significant. Therefore, to reduce the forming conditions as much as possible in actual sintering, we can select 70 MPa as the sintering pressure.

### 4.2. Effect of Sintering Temperature on the Densification of Inconel 718 Superalloy

To explore the effects of sintering temperature on the densification of the sintered sample, the sintering temperatures of 950 °C, 1050 °C, 1150 °C and 1250 °C were, respectively, applied to observe the changing trend of the porosity. The sintering process of superalloy powder is divided into two stages, namely, the heating stage and the holding stage. The influence of temperature on the sintering process is mainly reflected in the holding stage [28]. Therefore, to ensure the consistency of the initial conditions in the holding stage, the electric current density and the pressure rise rate should be adjusted separately so that the superalloy reaches the same large porosity after the end of the heating stage.

**Figure 5:**
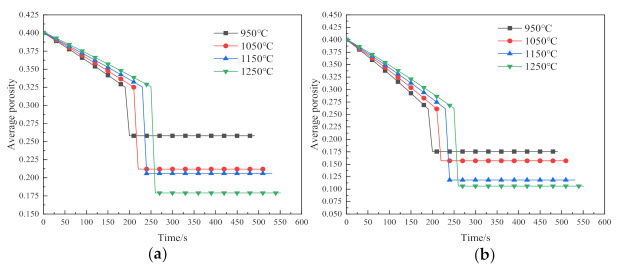

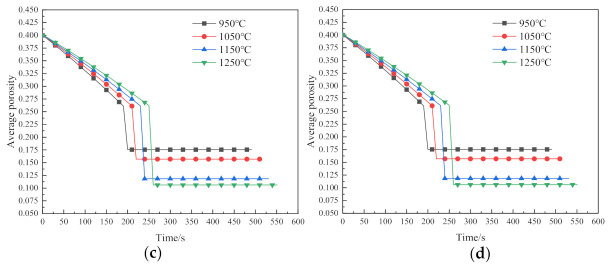
Effect of sintering temperature on porosity under various sintering pressures: (**a**) 50 MPa; (**b**) 70 MPa; (**c**) 90 MPa; (**d**) 110 MPa.

Figure 5 shows the relationship between average porosities of the sintered samples and the sintering time when the sintering temperatures are 950 °C, 1050 °C, 1150 °C and 1250 °C, respectively, under different pressure conditions. When the sintered sample reaches a certain degree of densification through heating and pressure increase and enters the holding stage, the porosities of the sintered samples at different temperatures all drops rapidly within 25 s, and the samples shrink sharply. At this stage, the boundaries between particles begin to fuse, forming the sintering necks [29] and the densification rate is correspondingly accelerated. After the rapid densification stage, the porosity reaches a steady state.

The higher the sintering temperature, the smaller the sintered porosity after reaching the steady state, and the better the degree of densification. The densification of sintering at 950 °C is mainly based on plastic flow, with the worst degree. The sintering densification at 950 °C is mainly based on plastic flow, and its degree of densification is the worst. The porosity at 950 °C drops to only 0.175 at the maximum pressure of 110 MPa, indicating that the temperature of 950 °C is not suitable as the experimental sintering temperature. It can be seen from Figure 5b–d, When the sintering temperature is 1150 °C and 1250 °C, the main driving force for densification is creep, and the average porosity difference after sintering is less than 0.1. Under the circumstance, the increase of the temperature has little effect on the porosity of the sintered sample. Therefore, 1150 °C can be selected as the experimental sintering temperature of Inconel 718. At 1150 °C, the densification rate is about 1.67 × 10^−3^ s^−1^. Compared with other advanced powder metallurgy methods, the densification rate has a great advantage for high forming efficiency.

### 4.3. Experimental Verification

The chemical composition of the Inconel 718 powder material used in this experiment is shown in Table 3.

**Figure 6:**
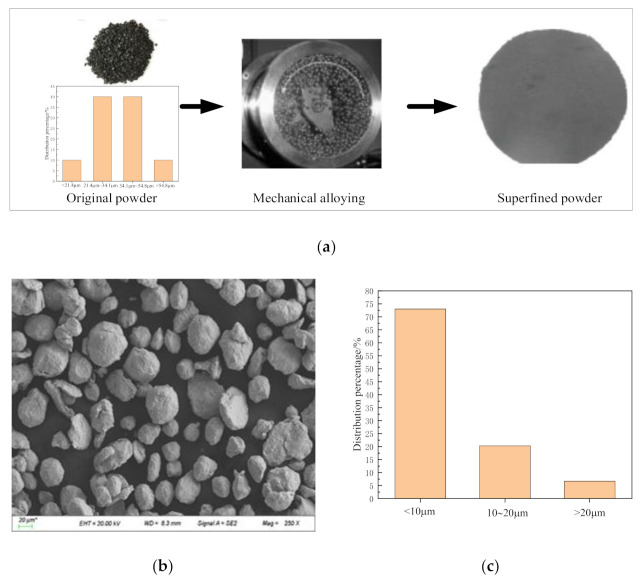
Original Inconel 718 superalloy powder and Inconel 718 powder and particle size distribution after ball milling: (**a**) original powders and alloying process; (**b**) Scanning electron microscope (SEM) image of powders after ball milling; (**c**) particle size distribution of Inconel 718 after ball milling.

Figure 6a presents the particle size distribution of the original powders. The diameters of 80% of the superalloy powder particles are between 21.4 and 54.8 μm, which are relatively large, failing to meet the requirements of mechanical properties after sintering. To reduce the particle sizes of the powders and enhance the uniformity of the sample structure obtained by sintering, the initial powders were ball milled by the method of mechanical alloying. Figure 6b shows the superalloy powders obtained after ball milling for 8 h, among which the proportion of the powders with diameters of less than 20 μm accounts for 65%, and the powders with the diameters between 20 μm and 30 μm account for 22% (as shown in Figure 6c).

**Figure 7:**
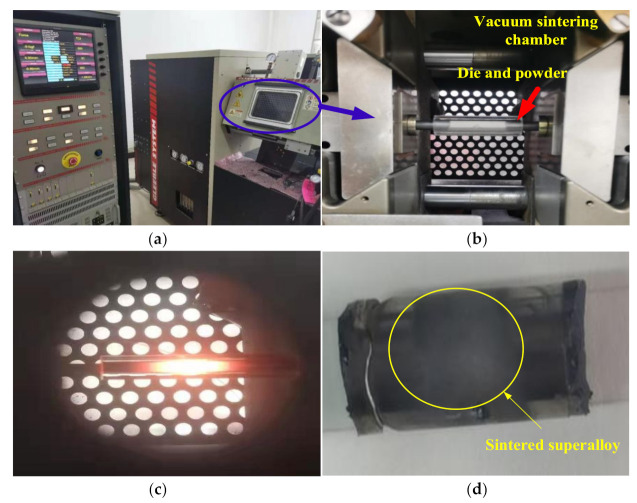
Experimental process and alloy sample after sintering: (**a**) vacuum sintering chamber; (**b**) vacuum sintering chamber and clamped mold and powder; (**c**) Inconel 718 alloy sample during sintering; (**d**) Inconel 718 alloy sample after sintering.

As indicated in Figure 7, the Inconel 718 powder sintering experiment process was conducted in Gleeble−1500 manufactured by Dynamic System Inc, USA. To measure the temperature in the sintering process, a through-hole with a diameter of 1mm was drilled in the middle of the quartz tube to locate the thermocouple. According to the analysis in Section 3.1 and Section 3.2, the sintering temperature of 1150 °C and the sintering pressure of 70 MPa were employed, and the holding time was 5min. The sintering process was divided into three stages: the heating stage, the holding stage and the cooling stage. The powder-added mold was clamped on the Gleeble-1500 (Figure 7a,b), and the vacuum was evacuated to 10^−3^ Pa. In 230 s, the temperature was raised to 1150 °C and the pressure was raised to 70 MPa. The temperature was held at 1150 °C and the pressure 70 MPa for 5 min (Figure 7c), so that the powders could be fully densified to form a uniform structure and then cooled in the furnace to obtain a densely sintered sample of Inconel 718 superalloy (Figure 7d). 

**Figure 8:**
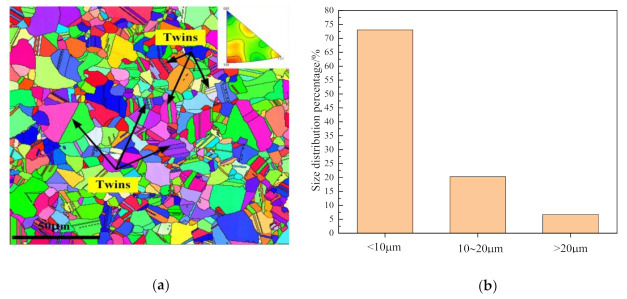
EBSD microstructure and grain distribution of the sintered sample: (**a**) EBSD microstructure of the sample after sintering; (**b**) grain distribution.

The density of the sintered sample is 7.8 g/cm^3^ measured through Archimedes principle, and the theoretical density of Inconel 718 is 8.24g/cm^3^, hence the relative density of the sintered sample is 94.46%. The sintered alloy sample was polished with sandpaper and argon ion, and the microstructure was observed by electron backscatter diffraction (EBSD). The detection interface is the transverse section of the sample. Figure 8 shows the EBSD microstructure and grain size distribution of the sintered sample. The grain orientation of the sintered sample is randomly distributed, and the proportion of twins in the grains of the sintered sample is calculated to reach 44.4% in Figure 8a. The Inconel 718 superalloy has a Face-Centered Cubic (FCC) structure, whose stacking fault energy is lower than other alloys (such as aluminum alloys). Therefore, it is easier to produce twin boundaries of twins after sintering and densification. The formation of twin boundaries proves that dynamic recrystallization occurs during the sintering process, which promotes the formation and expansion of the sintering neck, eliminates the pores between the grains, increases the relative density of the sintered sample and declares a good degree of densification, basically consistent with the numerical simulation results. In addition, the grain sizes in the figure were calculated as shown in Figure 8b. About 93% of the grain size is below 20 μm, and 73% of the grain size is below 10 μm, demonstrating that the electric field-assisted sintering also effectively controls the growth of grains, improves the uniformity of the structure and facilitates the formation of Inconel 718 superalloy with better mechanical properties.

**Figure 9:**
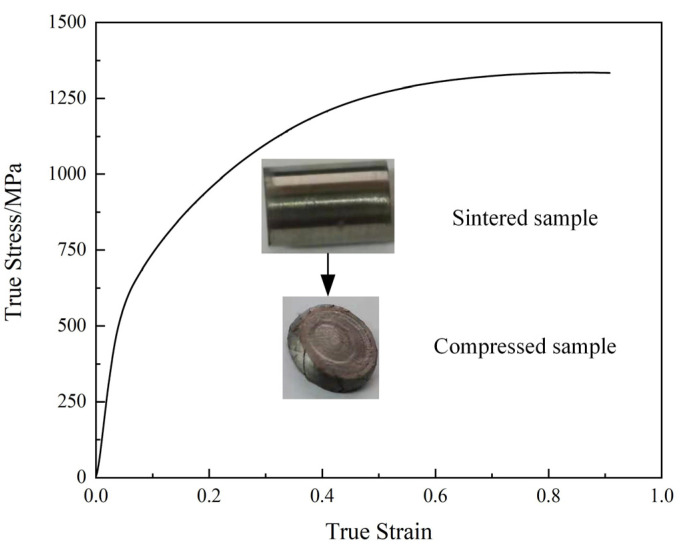
Mechanical properties of the sintered superalloy and the conventional superalloy via compressive testing.

To prove the advantage of the selected temperature and pressure of electric field-assisted sintering on the mechanical properties of Inconel 718 superalloy, the sintered sample was subjected to a unidirectional compression test on the MTS-LPS-204 universal testing machine (10 kN, MTS Industrial Co., Ltd., Eden Prairie, MN, USA) at a strain rate of 0.001 s^−1^ and a temperature of 20 °C, as shown in Figure 9. In addition, the microhardness of the sintered sample was measured with a Vickers hardness tester (HVT-1000 high-temperature vacuum hardness tester, Dongguan Hailiang Instrument Equipment Co., Ltd., Dongguan, China). The selected load was 5 kgf and the load holding time was 10 s.

Figure 9 shows the true stress-plastic strain of the sintered sample superalloy. The yield strength of sintered superalloy is 512 MPa, much higher than that sintered in Ref. [11], which is 419 MPa. In terms of compressive strength, the two values are both close to 1260 MPa, however, the deformation degree reaches 90% without fracture, and the compressive limit of sintered superalloy appears when the true strain is above 0.8. The above results all prove that the electric field-assisted sintering under the conditions of 1150 °C and 70 MPa can obtain Inconel 718 superalloys with superior mechanical properties, verifying the accuracy of the numerical simulation results.

When measuring the microhardness of the sintered sample, the average value was calculated within 10 measured points, mirroring that the average microhardness of the sintered sample was 395 Hv, while the microhardness of the sintered sample prepared in Ref. [11] was 300 Hv. It is seen that the electric field-assisted sintering can obtain a sample with desirable microhardness.

## 5. Conclusions

Based on the FKM-GTN model, a numerical simulation model for characterizing the electric field-assisted sintering process of Inconel 718 nickel-based superalloy was established, which realized the accurate description of the sintering process of Inconel 718 alloy under the coupling of electric-thermal-force. In addition, the effect of sintering pressure and temperature on alloy densification in the electric field-assisted sintering process was analyzed. The main conclusions were drawn as follows:

(1) When the sintering pressure is 50 MPa or below, the degree of densification is low regardless of the sintering temperature. When the pressure is over 110 MPa, the sintered sample presents the yield state prematurely due to rapid densification, and the sintering process is difficult to accurately control. When the sintering pressure is above 70 MPa, the densification cannot be significantly improved by changing the pressure.
(2) The main driving of the densification is plastic flow and the degree of densification is low when the sintering temperature is below 950 °C. When the temperature rises to 1150 °C, the sintered sample quickly completes plastic flow and enters a stage dominated by dislocation creep and diffusion creep and the densification reaches the desired level. When increasing the sintering temperature above 1150 °C, the average porosity of the sintered sample does not change much.
(3) Under the conditions of 70 MPa and 1150 °C, the sintered sample has a twin ratio of 44.4% and that the degree of densification is 94.46%, which consistent with the numerical simulation results. In addition, about 73% of the grain size is below 10 μm, effectively controlling the growth of the grains and conducive to the homogenization of the microstructure.
(4) The yield strength of the electronically sintered sample is 512 MPa, the deformation degree reaches 80% without fracture and the microhardess of the sintered sample is 395 Hv. The experimental results demonstrate an excellent mechanical property of the sintered sample.

## Figures and Tables

**Table 1:** Numerical simulation parameters of electric field-assisted sintering of Inconel 718.

Calculation Parameters	Numerical Value
Diameter of initial sintered sample *φ*/mm	9
Initial sintered sample length *l*/mm	50
Density of initial sintered sample *ρ*_p_/(kg·m^−3^)	3800
Young’s modulus of initial sintered sample *E*_p_/Gpa	3.3
Initial sintered sample Poisson’s Ratio νp	0.3
Thermal expansion coefficient of initial sintered sample *α*_p_/K^−1^	10^−7^
Thermal conductivity of initial sintered sample *k*_p_/(W·m^−1^·K^−1^)	30
Specific heat capacity of initial sintered sample at constant pressure *C*_p_/(J·kg^−1^·K^−1^)	450
Conductivity of initial sintered sample *λ*_p_/(S·m^−1^)	100
Yield stress of initial sintered sample *σ_s_*_p_/MPa	200
Porosity of initial sintered sample *φ*_0_	0.4
Tvergaard correction coefficient *q*_1_ Tvergaard correction coefficient *q*_2_ Tvergaard correction coefficient *q*_3_	1.5 1 2.25
Die density *ρ*_d_/(kg·m^−3^)	2600
Young’s modulus of die *E*_d_/Gpa	60
Poisson’s ratio of die *ν*_d_	0.25
Die thermal expansion coefficient *α*_d_/K^−1^	7 × 10^−6^
Die heat transfer coefficient *k*_d_/(W·m^−1^·K^−1^)	129
Die constant pressure specific heat capacity *C*_d_/(J·kg ^−1^·K^−1^)	710
Die conductivity *λ*_d_/(S·m^−1^)	10,000
Relative permittivity *e*	1

**Table 2:** Numerical simulation program.

Serial Number	Sintering Temperature (°C)	Sintering Pressure (MPa)
1	1250	50
2	1250	70
3	1250	90
4	1250	110
5	1150	50
6	1150	70
7	1150	90
8	1150	110
9	1050	50
10	1050	70
11	1050	90
12	1050	110
13	950	50
14	950	70
15	950	90
16	950	110

**Table 3:** Chemical composition of Inconel 718 powder (wt.%).

Element	Quality Ratio
Ni	52.17
Nb	5.24
Mo	3.13
Cr	19.02
Al	0.41
Ti	0.89
Co	0.0089
B	<0.005
Si	0.06
Mn	<0.005
Cu	0.091
Mg C S	<0.005 0.022 <0.003
P	0.056
Fe	rel.

## Data Availability

The data presented in this study are available on request from the corresponding author.
